# Spatial Distribution and Pollution Level of Heavy Metals in Street Dust of the City of Suwałki (Poland)

**DOI:** 10.3390/ijerph20064687

**Published:** 2023-03-07

**Authors:** Mirosław Skorbiłowicz, Łukasz Trybułowski, Elżbieta Skorbiłowicz

**Affiliations:** Department of Technology in Environmental Engineering, Faculty of Civil Engineering and Environmental Sciences, Bialystok University of Technology, Wiejska 45E, 15-351 Białystok, Poland

**Keywords:** environmental studies, street dust, heavy metals, pollution assessment, spatial distribution, pollution source identification

## Abstract

This paper presents an analysis of the content and spatial distribution of heavy metals (HM) in street dust in Suwałki, a city located in northeastern Poland. The HM content of street dust was also evaluated using the geochemical index (Igeo), enrichment factor (EF), and contamination factor (CF), and local HM sources were identified using chemometric methods. The arithmetic averages of HM contents in dust arranged in the following order: Fe > Zn > Mn > Cu > Cr > Ni > Pb, were 11,692.80, 215.97, 194.78, 142.84, 63.59, 17.50, 17.04 mg∙kg^−1^, respectively. Higher values than the local background occurred for Cr, Cu, Ni, Zn and Pb. The values of Igeo, CF, and EF indicate that the highest pollution in dust is due to Zn and Cu. The spatial distribution of metals was evaluated using maps of HM content in road dust samples from Suwałki. The spatial distribution of HM showed areas with high contents of Cr, Cu, Ni, Zn and Pb located mainly in the central and eastern parts of the city. In these areas, high traffic volume, the presence of shopping centers, administrative buildings and bus stops dominate. Statistical models of multivariate analysis (FA) and cluster analysis (CA) identified two sources of HM. The first source of pollution was associated with local industrial activity and motor vehicle travel, and the second with natural sources.

## 1. Introduction

Increased industrialization and progressive urbanization around the world are contributing to the excessive occurrence of heavy metals (HMs) in the environment [1]. The intensive development of the road transport sector and industry largely contribute to the continuous increase in concentrations of these metals in road dust. Roads play a significant role in social life and economic development; moreover, HM-contaminated road dust is an increasing environmental problem due to its impact on human health and ecosystems [2]. Dust accumulated on the road surface is a composite of natural deposits, including soil material, organic matter, and atmospheric particles and anthropogenic deposits, resulting from the degradation of vehicle components, combustion products, road paints, road salt used in winter, and construction materials [3]. Dust particles are subjected to many complex mixing processes that occur on the street surface. Therefore, the composition of street dust can vary from place to place. In addition, street dust contains HMs from ground or overhead lines used by streetcars, trolleybuses, and trains [4]. Road surfaces make up only a small part of the surrounding landscape; however, their high degree of impermeability significantly increases the proportion of HMs generated into the environment [5,6]. In addition, accumulated dust during dry weather can easily migrate as a result of turbulence caused by motor vehicle traffic and atmospheric conditions, which can consequently significantly affect the quality of air, soil, surrounding vegetation and people [7,8]. In urbanized areas, particularly cities, studies have shown that the main anthropogenic sources of HM are traffic, emissions from industrial processes, and agricultural activities [2]. With the technological progress of society, the contribution of pollutants to street dust continues to change. The advent of hybrid and electric vehicles will lead to a reduction in emissions, but may also increase the concentration of non-hybrid emissions [9].

The HM content of road dust varies widely depending on traffic and road features (traffic circles, highways, traffic lights, etc.). During high-speed braking, the brakes are exposed to extensive heat from friction, which is transferred to the brake discs and causes particle emissions. The most intense brake wear obviously occurs at intersections, road curves, traffic lights and forced braking [10]. They estimate that about 50% of the residue from worn brakes is deposited on or near roads. Road dust can also become contaminated through the abrasion of the tire and road surface. According to Panko et al. [11], between 0.1 and 10% of tire residue becomes airborne. The rest is deposited on or in close proximity to roads. People living near roads or using sidewalks in urban areas are most exposed to HMs accumulated in road dust and other traffic-related pollutants [12].

The subject of this paper is a traffic dust analysis based on material collected in 2019 in Suwałki, a city located in the northeastern part of Poland. The city’s high natural and cultural attractiveness influences the increase in traffic during the summer. The city plays the role of a link between the various tourist sub-areas and attractions of the region. A characteristic feature of the city is the location of urbanized areas along Gen. K. Pułaski Street and Utrata Street, which marks the axis of national road No. 8 running from the Czech border in Kudowa-Zdroj to the Lithuanian border in Budzisko, along which more than 3 million vehicles travel annually.

The objectives of this study were: (1) to determine the content of HMs (Zn, Cu, Pb, Ni, Cr, Mn, Fe) in street dust; (2) to assess the degree of heavy metal contamination with the following indices: geochemical index (Igeo), enrichment factor (EF), and contamination factor (CF); (3) to analyze the spatial distribution of heavy-metal content in street dust, and (4) to identify the sources of contamination using chemometric methods and to identify the governing factors of HMs and their distribution trends in street dust.

This article is the first study of HM in street dust in Suwałki, thereby contributing to knowledge, better management of this city in the future, and also will support the development of government strategies to combat environmental pollution.

## 2. Materials and Methods

### 2.1. Research Area

The city of Suwałki (22°52′ and 23° 00′) is located in the northeastern part of Poland, occupying an area of 66 km^2^ and a population of about 70,000. It is located along important transportation routes, including national road No. 8, which is part of the international route E67, and provincial roads. The route of national road No. 8 runs in a corridor located to the east of the city’s inner center, while in the northeastern area it crosses areas of high residential development. Intense automobile traffic, especially transit truck traffic passing through the city, is a major cause of air pollution, as well as a source of noise and traffic stress for city residents. In April 2019, an approximately 13-km section of the Suwałki bypass was put into service. Prior to the construction of this route, all heavy vehicles passed through the main street of the city heading towards the state border with Lithuania. With more than 8000 a day and 3 million trucks a year passing through the city, the amount of traffic on the city’s main street is counted among the largest in the country. The specific location and the lack of urban centers of similar size within a radius of several dozen kilometers make the city the main administrative, service, and cultural center of the subregion. The agricultural nature of the county as a producer of raw materials for the agri-food industry assigns a decisive role to this industry among other sectors of the economy. Agri-food processing is based on the local raw material base. The larger, leading buyer of milk from Suwałki is the “SUDOWIA” Dairy Cooperative in Suwałki. An important role in agri-food processing is also played by “Animex Grupa Drobiarska” Sp. z o.o. in Suwałki, which obtains chickens and geese from the Suwałki District. Suwałki is also a center for the timber and building materials industries, among others. Sources of metals in the Suwałki area include sections of roads with heavy traffic and areas in the vicinity of gas stations. Other significant sources of metals include the burning of hard coal. Hard coal is used as fuel for thermal power plants; this material is also an important raw material for heating homes. Its combustion causes the release of large amounts of dust pollution containing various heavy metals, and the elements released in this way can pollute the city’s environment. Within the city’s administrative boundaries there is a nature reserve and areas of protected landscape, along with naturally and historically valuable areas around the city. Suwałki is located in a temperate climate zone with clearly marked by “continentalism”, and temperatures in the city and its environs are among the lowest among the lowlands of Poland. The average multi-year annual temperature is 6.8 °C, while the annual temperature amplitude is 22.9 °C. The warmest months are July and August, while the coldest months are December, January, and February. Winds from westerly directions predominate, their speeds rarely exceeding 5 m/s. They blow the strongest in March and November. It is also then (unlike in other seasons) that eastern and southeastern winds occur with greater frequency. The number of days with precipitation, however, is lower than in other regions, indicating significant rainfall.

### 2.2. Sample Collection and Preparation

**Figure 1:**
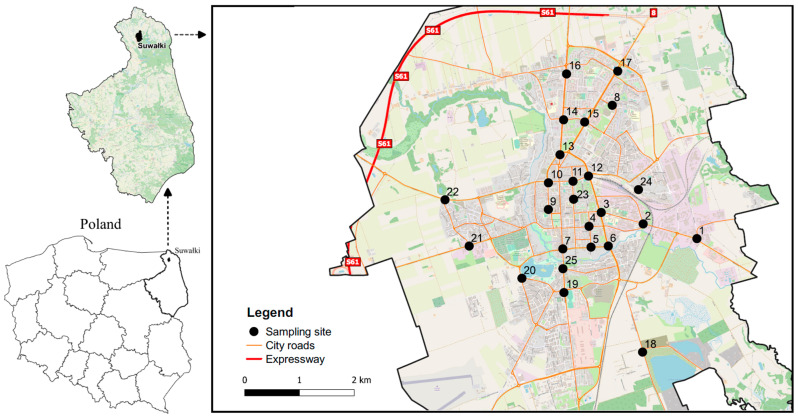
Location of the study area with 25 measurement points within the city of Suwałki.

Twenty-five street dust sampling sites were selected in the urban area (Figure 1). In order to obtain samples with a certain representativeness of a given sampling point, the current city plan was carefully analyzed. For this purpose, the city plan was analyzed with the help of QGIS software, enabling cartographic data management. In addition to a cartographic analysis of the city, field surveys were conducted. Sampling sites were closely analyzed both in terms of traffic intensity and the location of industrial plants within the given sampling points. A smaller number of sampling points occurred in the western part of the city because it is mainly single-family housing, with relatively low traffic intensity, and no industrial plants. Such a method of determining sampling sites was justified so that the obtained test results will be compared with the sampling site, which will make it possible to determine the factors shaping the formation of heavy metals at a given sampling point.

Road dust sampling was conducted in the spring during the dry weather period in 2019. The length of the dry period preceding sampling affects the granulometric composition and concentration of pollutants [8], so sampling was done after at least 3 days of dry weather. The material for the study was taken from both sides of the roadway lane adjacent to its edge where the accumulation of street dust is greatest. The area sampled was one square meter (1 m^2^). Samples were collected by sweeping about 500 g of material with a brush and a clean plastic dustpan into plastic self-sealing polyethylene bags and transported to the laboratory. In order to avoid re-hanging the finest particles, the sampling process was carried out very slowly. All samples were pre-dried for a minimum of 10 days at room temperature, about 20 °C. The dried samples were passed through a nylon sieve with a fraction of 1 mm to remove foreign matter such as small pieces of paving stones, leaves, glass and other impurities, and then dried in porcelain crucibles at 105 °C using an induction furnace. The samples were then passed through a nylon sieve with a 200 µm fraction to extract the base fraction for further testing. The dried samples were subjected to grinding in an automatic agate mortar. All sampling and handling procedures were carried out without contact with metals to avoid potential contamination.

### 2.3. Analytical Methods

Street dust of 1.0 g was mineralized with HNO_3_ with the addition of 30% H_2_O_2_ (catalog no. A.C.S-118851934) as an oxidant in a closed microwave system. Extraction of elements into solution was performed according to the US Environmental Protection Agency’s method 3051A. The digestion process was carried out under the following conditions: Step 1: 20 min, 210 °C, 1800 W; Step 2: 15 min, 210 °C, 1800 W. All determinations were performed in triplicate. Samples were quantitatively transferred to 50 mL volumetric flasks after filtration. The contents of Cr, Cu, Ni, Zn, Pb, Fe, Mn were determined by flame atomic absorption spectrometry (AAS) on an AAS ICE 3500 Thermo Scientific spectrometer. All solutions were prepared using ultrapure water. Glassware that was used for the determinations was soaked in nitric acid (5%) and washed with tap water, followed by a thorough rinse with deionized water. The results of the street dust analyses were verified using certified reference material Certificate No. 0217-CM-7003. The measurement results of the standard reference material showed good agreement with the certified values. The results obtained for the content of the tested elements were given for air-dry dust. The reaction was measured potentiometrically in a dust:water suspension (1:2.5) using a pH meter. Organic matter (OM) content was measured as mass loss after roasting at 450 °C.

### 2.4. Statistical Analysis

All statistical analyses were performed using the licensed program Statistica ver 13.3 for Windows. The Shapiro–Wilk test, which is optimal for the number of data (n = 25), was used to verify the normal distribution. The results were considered statistically significant with a probability of error of *p* < 0.05. Most of the data did not have a distribution close to normal, subjected to transformation. The data were then subjected to further statistical analysis. Pearson’s correlation analysis was used to examine the relationships between HMs in road dust and identify their sources. The correlation coefficient was used to measure the interrelationships between the two metals. Multivariate statistical analysis (PCA), which is often used for environmental studies, was also used for analysis [13,14,15]. Before the principal component analysis (PCA), the KMO (Kaiser–Meyer–Olkin) index and Bartlett’s sphericity test were performed. The KMO value was obtained in the range from 0.55 to 0.80 and Bartlett’s test was statistically significant. Ward’s version of statistical cluster analysis (CA) was also used to classify metals from different sources but with similar physical and chemical properties [16].

### 2.5. Assessment of Heavy Metal Pollution

In order to quantify the degree of HM contamination and determine the degree of enrichment of street dust in heavy metals, three of the most commonly used indices were used: the geochemical index (Igeo), the enrichment factor (EF), and the contamination factor (CF) [4,17]. Equation (1) was used to calculate Igeo [18]:

(1) $$I_{\mathrm{geo}}=\log_{2}\left(\frac{C_{n}}{1.5\cdot B_{n}}\right)$$

where C_n_ is the measured concentration of the test metal in street dust (mg·kg^−1^), B_n_ is the geochemical background concentrations by Czarnowska (1996), while 1.5 is the constant correlation coefficient of the background matrix due to lithogenic variability [19,20,21]. The geoaccumulation index (Igeo) can be divided into seven classes, which are as follows: uncontaminated, class 0 (Igeo ≤ 0); uncontaminated or moderately contaminated, class 1 (0 < Igeo < 1); moderately contaminated, class 2 (1 < Igeo < 2); moderately or heavily contaminated, class 3 (2 < Igeo < 3); heavily polluted, class 4 (3 < Igeo < 4); heavily to very heavily polluted, class 5 (4 < Igeo < 5); very heavily polluted, class 6 (Igeo ≥ 5) [18].

To assess heavy metal contamination of road dust and the likely contribution of anthropogenic sources, (EF) was used [22,23]. In order to minimize the bias caused by heterogeneous samples, a reference element is used. Fe was used as the reference element. EF is calculated for each element individually according to Equation (2) [24]:

(2) $$\mathrm{EF}=\frac{{\left(C_{n}/C_{\mathrm{ref}}\right)}_{\mathrm{Sample}}}{{\left(B_{n}/B_{\mathrm{ref}}\right)}_{\mathrm{Background}}}$$

where C_n_ is the mass concentration of the target component, C_ref_ is the concentration of the selected reference component, B_n_ geochemical concentration of the target component by Czarnowska (1996), while B_ref_ is geochemical concentration of the reference component by Czarnowska (1996). The degree of elemental contamination can be divided into five categories [25]: (1) EF < 2, minimal enrichment; (2) 2 ≤ EF < 5, moderate enrichment; (3) 5 ≤ EF < 20, significant enrichment; (4) 20 ≤ EF < 40, very high enrichment; and (5) EF ≥ 40, extremely high enrichment. EF values greater than 10 indicate anthropogenic sources of metals, while EF values less than 10 indicate the origin of metals mainly from the underlying soil.

CF is used to determine the level of HM contamination. It is defined by Equation (3) [26]:

(3) $$\mathrm{CF}=\frac{{\left(C_{m}\right)}_{\mathrm{Sample}}}{{\left(C_{m}\right)}_{\mathrm{Background}}}$$

It is calculated as the ratio of the concentration of the studied n-element to its concentration in the earth’s cluster (geochemical background). Contaminants are classified according to a four-level scale [26,27]: CF < 1, low contamination factor; 1 ≤ CF < 3, medium contamination factor; 3 ≤ CF < 6, significant contamination factor; CF ≥ 6, very high contamination factor.

### 2.6. Spatial Analysis

Maps of the spatial characteristics of HM within the city were made using the licensed program Surfer 22 license no: 687-111-SSE. The Kriging method commonly used by other researchers [28,29] was used to determine a regular grid of values based on geostatistical methods. Kriging is an advanced geostatistics method based on a linear variogram model. A variogram is characterized by the spatial continuity of a data set, and is also called a semivariogram. The idea of the variogram assumes that function values in close proximity are highly correlated, it takes into account not only distance dependence, but also directional dependence. The variogram model is the basis of the interpolation procedure, which results in the determination of a regular grid of values. Conducting analyses of the spatial distribution of metals in street dust on urban roads is helpful in identifying sites with elevated metal content, as well as in assessing potential sources of pollution.

## 3. Results and Discussion

Table 1 shows descriptive statistics of the HM studied and other parameters of road dust. The factors affecting the amount of HM in road dust are organic matter and reaction. The average OM content in the dust was 2.24% and ranged from 0.70 to 7.90% and its variability (CV) was at a high level of 68.33%. The high variability of OM content suggests mixed source impacts [30]. The contents found are similar to those obtained by Robertson et al. [31], Shilton et al. [32], and O’Shea et al. [33]. The reaction (pH) of the dusts tested in most samples was alkaline, ranging from 7.31 to 8.77. High pH values promote adsorption and precipitation, while low pH can weaken the bonding force and hinder the retention of metals in the dusts.

The arithmetic averages of the HM contents in the dust arranged in the following order: Fe > Zn > Mn > Cu > Cr > Ni > Pb, and were 11,692.80, 215.97, 194.78, 142.84, 63.59, 17.50, 17.04 mg∙kg^−1^, respectively. The local background proposed by Czarnowska [34] and the geochemical background established by Taylor and Mclennan [35], i.e., the content of elements found in the Earth’s crust, were used to evaluate dust pollution. The analyses carried out showed elevated average contents of Cr, Cu, Ni, Zn, Pb, while the contents of Fe and Mn turned out to be slightly lower, with no obvious enrichment with respect to the background values. The maximum contents of Cr, Cu, Ni, Zn, Pb were 102.21, while 366.05, 41.56, 325.26, 47.32 mg∙kg^−1^ were 4-, 52-, 4-, 11-, 5-times higher than the local background values. This may indicate the anthropogenic origin of the metals studied. The study showed high variability (CV) of Pb (68.57%), moderate Cu (55.63%) and Mn (52.22%), and low variability of Cr (26.54%), Ni (33.80%), Zn (21.31%) and Fe (19.96%). The high variability of Pb and Cu indicate their heterogeneity in the environment, which may indicate their anthropogenic origin [36,37]. Most metal contents except Cr, Zn, and Fe do not follow a normal distribution as indicated by *p*-values (Shapiro–Wilk test). Zhang et al. [38] states that this is natural for geochemical variables. Fe, Zn, and Mn were recorded most in the dusts studied, while Pb was recorded least, which is largely dictated by the geochemical properties of these elements. De Andrade et al. [39] state that elements such as Fe and Mn are found in large quantities in regional soils and come from natural sources such as the Earth’s crust. Both metals may come from the weathering of rock fragments rich in these elements. Fe in road dust may also enter the environment from activities such as the use of vehicle brake pads, and Mn comes mainly from vehicles running on unleaded gasoline, the Pb compounds once added to gasoline having been converted to Mn and, hence, its increased emissions. Adamiec et al. [10] indicates that large amounts of emitted Zn are produced by tire abrasion. Smolders and Degryse [40] claims that tires contain up to 4.3% Zn, plus it can come from abrasion of traffic signals and barriers. Zinc compounds also act as antioxidants for oil in the combustion chamber [41]. When analyzing Cu content in road dust, it is worth noting that it is a common element in automobile bearings, brake linings, and other engine parts [42]. Brass and Cu are components of automotive radiators due to their high corrosion resistance and high thermal conductivity [12]. The metal also seeps into the environment as a result of wear on the automotive oil pump or corrosion of metal parts in contact with oil [43]. Rajaram et al. [2] state that Zn and Cu are also indicators of metal emissions from non-combustion sources and their high levels confirm the presence of these elements in many countries. Very similar results and, in particular, the highest levels of Zn and Cu contamination in the dust of Warsaw were obtained by Dytłow and Górka-Kostrubiec [44] and in Krakow, Katowice, and Olkusz [22]. The studies conducted showed low contents of Ni and Pb in road dust. Ni and Cr in road dust can come from marking paint of anti-corrosion coatings on vehicles and from friction processes of tires and roadway surfaces, as well as safety barriers. Numerous studies conducted to date indicate that anthropogenic sources of potential toxic metals in road dust are mainly traffic emissions (vehicle exhaust particles, tire wear particles, weathered street pavement particles, brake lining wear particles) and industrial sources (power plants, coal burning) [45,46]. As shown in Table 2, the HM content of Suwałki’s road dust is lower and comparable and even in some cases higher than its content in dust from other cities in Poland and the world. Suwałki’s population is not comparable to most cities in the table. This may be related to the large transit traffic of vehicles heading toward the Polish–Lithuanian border.

### 3.1. Assessment of Heavy Metal Pollution of Road Dusts

**Figure 2:**
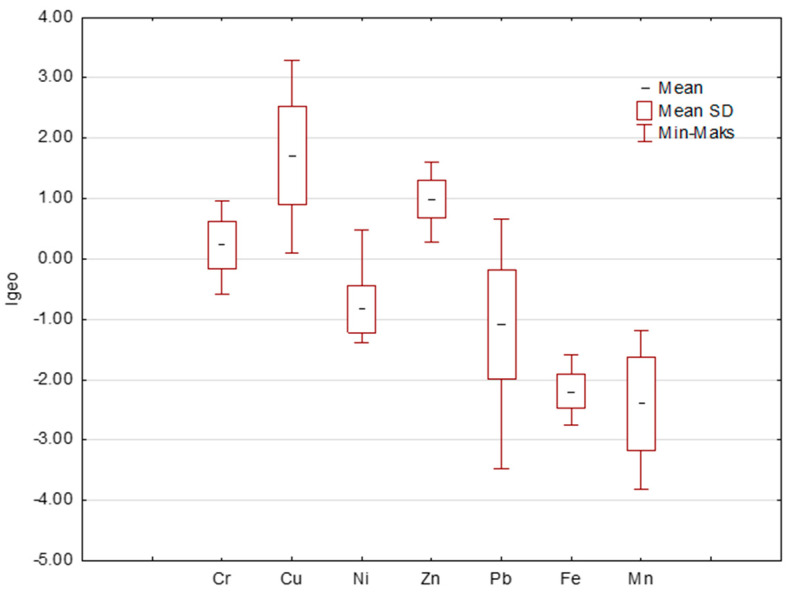
Assessment of level of pollution by Igeo.

The geoaccumulation index (Igeo) [12,52] provides useful information about the level of pollution. The average Igeo values were in the following order: Mn < Fe < Ni < Pb < Cr < Zn < Cu. All values of this index for elements such as Ni, Pb, Fe and Mn allowed for the classification of the tested dusts into class 0, i.e., as uncontaminated with the above metals. On the other hand, the average values of Cr (0.23) and Zn (0.99) falling within the range of 0 ≤ Igeo ≤ 1 indicated an uncontaminated or moderately contaminated level. The highest level of contamination falling within the range of 1 ≤ Igeo ≤ 2 was obtained for Cu (1.72), as moderately contaminated [18]. Figure 2 presents the values of the Igeo index.

**Figure 3:**
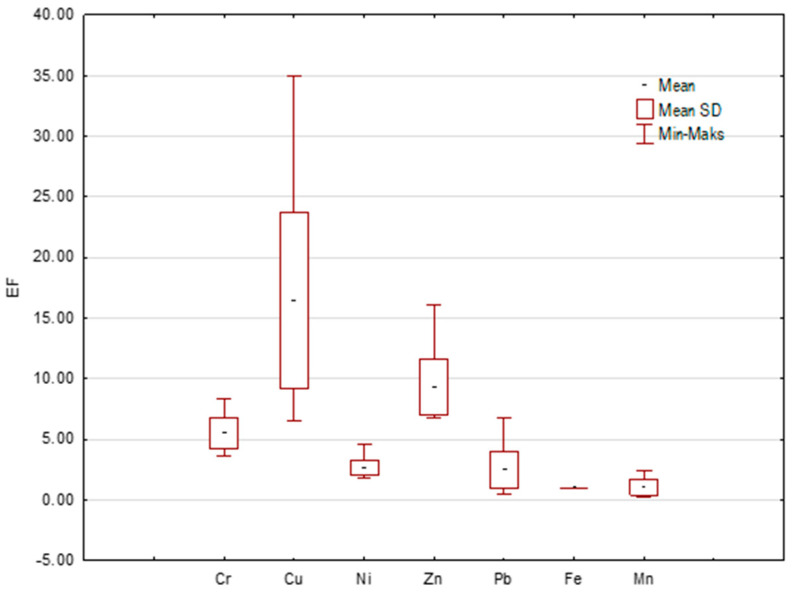
Assessment of level of pollution by enrichment factor (EF).

An enrichment factor (EF) can be used to assess metal contamination of road dust and the likely contribution of anthropogenic sources [36,53,54]. Slightly different results were obtained by analyzing the values of the EF index than the Igeo values. The values of this index for Fe and Mn allowed us to classify the studied dusts as minimal enrichment. The average EF values for Ni and Pb ranged from 2 to 5, which means moderate enrichment of dusts in these elements. Significant enrichment was shown by Cu, Zn, and Cr for which the EF values ranged up to 5 to 20 which suggests significant enrichment having an eminently anthropogenic origin. Figure 3 shows the EF values.

**Figure 4:**
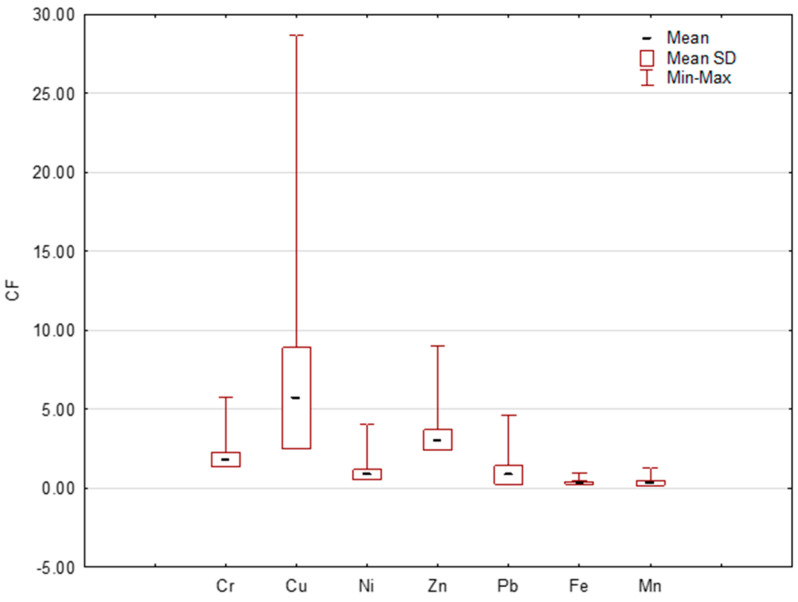
Assessment of level of pollution by contamination factor (CF).

They applied the contamination factor (CF) to their calculations [26,27,55] as the level of contamination caused by individual HMs. The average CF values for Ni, Pb, Fe and Mn were <1, indicating a low level of metal pollution, and only moderate CF > 1 values for Cr were of anthropogenic origin. High levels of CF contamination falling in the range of 3 ≤ CF < 6 were shown for average Cu and Zn contents. The maximum CF value for Cu reached an extreme level (29) as very high pollution (CF > 6). Very similar CF results were obtained by Dytłow and Gorka-Kostrubiec [44]. The CF values are shown in Figure 4.

### 3.2. Spatial Distribution of Metals in Road Dust

**Figure 5:**
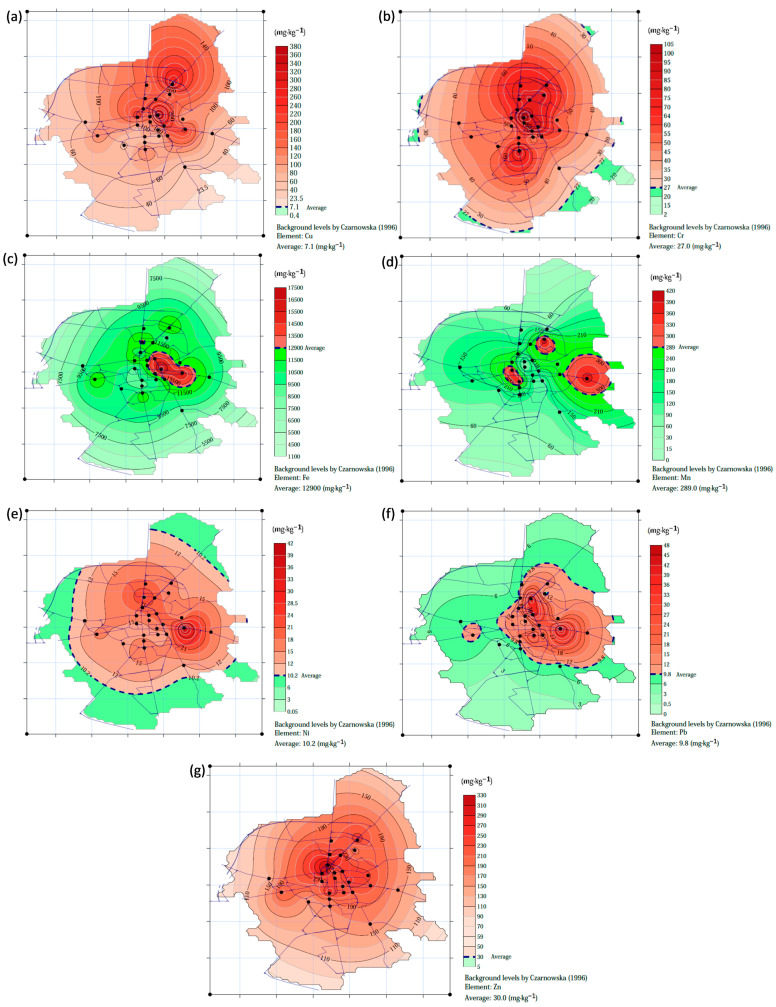
Spatial distribution of heavy metals in road dust in Suwałki: (**a**) Cu, (**b**) Cr, (**c**) Fe, (**d**) Mn, (**e**) Ni, (**f**) Pb, (**g**) Zn [34].

Spatial distribution analysis is helpful in identifying contaminated and uncontaminated zones in a given area. The spatial distribution of Cr, Cu, Ni, Zn, Pb, Fe and Mn in road dust is shown in Figure 5. The presence of most metals is mainly related to the wear processes of motor vehicle tires and brake pads. Areas with high contents of Cr, Cu, Ni, Zn and Pb are mainly located in the central and eastern parts of the city, and smaller ones in the western part. The zone of higher Pb contents is heavily confined to the central and eastern parts of the city. Presumably, this is the result of the gradual reduction of Pb in the environment as a result of the use of unleaded gasoline to power motor vehicles. The central zone of the city is dominated by heavy traffic, which is associated with the presence of shopping centers, administrative buildings, bus stops, etc. A different spatial distribution was shown for Mn and Fe. In most of the city’s area, the content of Fe and Mn oscillates below background, while small areas are visible where this level is exceeded. These areas are mostly found in the eastern part of Suwałki. The spatial distributions of Mn and Fe are more concentrated in comparison with the other metals. Outside of these areas, there is less traffic and inhabited areas, where a higher proportion of cultivated land of different nature is evident. Therefore, Fe and Mn in these areas may be partly of geogenic origin due to resuspension of soil material, among other factors.

### 3.3. Identification of Pollution Sources Using Statistical Analysis

Pearson’s correlation coefficients for metals in road dust are shown in Table 3. An analysis of correlation coefficients was carried out to assess the relationship between components of road dust. Positive average correlations between metals in Cr-Cu-Ni-Zn-Pb-Fe dust (r > 0.40) suggest that the main common origin is anthropogenic industrial activity. Studies indicate that the origin of Cu and Zn is related to wear and tear of internal combustion engine components [1,53,56]. The origin of Zn is associated with abrasion processes of car tires, brake wear and corrosion of vehicle components, and road infrastructure [57]. Similar correlations have been shown and suggested by the common source of the metals studied [2]. A correlation coefficient of less than 0.2 was considered insignificant [30]. Cr and Cu correlated with motor vehicle traffic volume at r > 0.50, which explains their direct origin. It turned out that pH had little effect on metals in road dust. This is indicated by weak pH-metal correlations. Organic matter only in one case (OM-Mn, r = 0.46) could affect the formation of Mn in dust.

A factor analysis was conducted in which two factors together explained 54% of the total variance (35% and 19%). Factor one (35%) correlated with Cr, Cu, Ni, Zn Pb and Fe. It was associated with local industrial activity and the movement of motor vehicles. Metals correlated with factor one also correlated with each other, indicating a similar source of origin. Similar conclusions were reached by Jeong et al. [58] but their study involved a city in South Korea. They too argue that there should be regular road cleaning in cities and industrial regions, which is not always the case. The second factor explained 19% of the total variance and correlated with Mn. The origin of Mn is related to motor vehicle traffic and its natural sources. Mn correlated with OM (organic matter), however, which may indicate its natural origin. Large natural Mn contents are found in soils. The rotated component matrix for the metals studied in the city of Suwałki is shown in Table 4.

**Figure 6:**
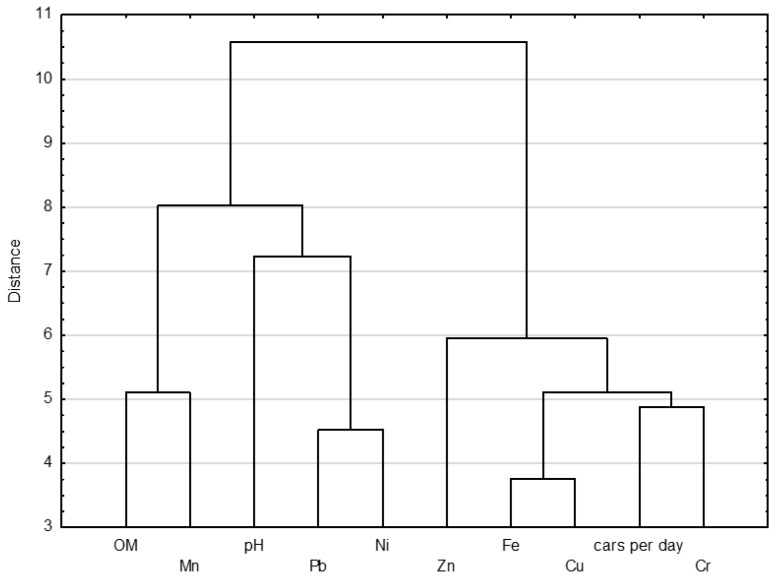
Hierarchical dendrograms for heavy metals in dust obtained by Ward’s hierarchical clustering method.

In this study, Ward’s version of cluster analysis was applied based on the measurement of Euclidean distance of similarities [59]. Figure 6 distinguishes two groups: group 1 (OM and Mn, pH, Pb and Ni) and group 2 (Zn, Fe, Cu, cars per day and Cr). Group one identifies natural and historical sources and group three represents traffic-related sources. The classification of metals was mostly dependent on their content, which had its own range for each class. The results achieved are consistent with the FA analysis.

## 4. Conclusions

The analytical results presented in this article have led to the following specific conclusions:

- Arithmetic averages of HM contents in dust arranged in the following order: Fe > Zn > Mn > Cu > Cr > Ni > Pb, and higher values than local background occurred for Cr, Cu, Ni, Zn, Pb;
- Values of Igeo, CF, and EF indicate that the highest pollution in dust is due to Zn and Cu are indicators of emissions from non-combustion sources and their high levels confirm the presence of these elements in many countries;
- Spatial distribution analysis indicated areas with high levels of Cr, Cu, Ni, Zn and Pb are mainly located in the central and eastern parts of the city where heavy traffic and the presence of commercial and administrative centers, bus stops dominate. The spatial distribution of Mn and Fe is different, these areas are mostly found in the eastern part of Suwałki where there is less traffic and a higher proportion of cultivated land of different nature is visible. Consequently, Fe and Mn may be partly of geogenic origin in these areas;
- Factor analysis identified two factors. The first factor correlated with Cr, Cu, Ni, Zn Pb and Fe was related to local industrial activities and the movement of motor vehicles. The second factor correlated with Mn and OM. The origin of Mn is related to motor vehicle traffic and its natural sources. The results of FA analysis were confirmed by CA analysis;
- As shown, HM contents in Suwalki road dust are lower and comparable and even in some cases higher than their contents in dust from other cities in Poland and the world. The study indicates that the sources of metals in road dust are mainly traffic emissions (car exhaust particles, tire wear particles, weathered street pavement particles, brake lining wear particles) and industrial sources (power plants, coal combustion);
- It should be emphasized that it seems advisable to introduce analysis of heavy metal content in road dust into routine monitoring studies. The research results obtained would allow the development and constant updating of guidelines for the strategy of organizing transportation in cities and the introduction, for example, of so-called low-emission zones, the operation of which has proved very successful in Stockholm, Berlin, and London, among others. Another way to reduce the migration of heavy metals is in the use of phytoremediation techniques, specifically plants referred to as hyperaccumulators. Vegetation separating roadways from sidewalks will largely contribute to the creation of natural barriers that reduce road dust emissions into the urban environment.

## Figures and Tables

**Table 1:** Descriptive statistics of metal concentrations (mg∙kg^−1^) in Suwałki road dust.

Parameter	N	G.b. ^1^	G.b. ^2^	Average	Min	Max	SD	CV [%]	Shapiro–Wilk Test
Cr	25	27.00	35.00	63.59	35.06	102.21	16.88	26.54	0.53
Cu	25	7.10	25.00	142.84	40.39	366.05	79.46	55.63	0.04
Ni	25	10.20	20.00	17.50	11.46	41.56	5.92	33.80	0.00
Zn	25	30.00	71.00	215.97	129.20	325.26	46.02	21.31	0.97
Pb	25	9.80	20.00	17.04	2.71	47.32	11.69	68.57	0.00
Fe	25	12,900.00	35,000.00	11,692.80	7828.72	17,393.52	2333.38	19.96	0.54
Mn	25	289.00	600.00	194.78	63.68	397.33	101.71	52.22	0.02
OM	25	-	-	2.24	0.70	7.90	1.53	68.33	0.00
pH	25	-	-	-	7.31	8.77	-	-	
cars per day	25	-	-	3328.72	650.00	8358.00	2172.59	65.27	0.01

^1^ Geochemical background by Czarnowska (1996) [34]; ^2^ geochemical background by Taylor and McLennan (1985) [35].

**Table 2:** Average concentration (mg·kg^−1^) of metals in road dust from various cities in the world.

City	Population	Zn	Cu	Pb	Ni	Cr	Mn	Fe	Reference
Birmingham (Anglia)	2300,000.00	534.00	466.90	48.00	41.10	-	-	-	[1]
Madrid (Hiszpamia)	2,909,792.00	476.00	188.00	1927.00	44.00	61.00	362.00	19,300.00	[47]
Kavala (Grecja)	54,027.00	272.00	124.00	301.00	58.00	196.00	-	-	[16]
Ottawa (Kanada)	934,240.00	101.00	38.10	33.50	14.80	41.70	-	-	[48]
Lublin (Polska)	341,975.00	201.80	65.70	23.30	26.80	52.80	-	-	[49]
Warszawa (Polska)	1,735,000.00	63.60	30.60	33.85	10.31	-	134.10	600.00	[50]
Białystok (Polska)	294,153.00	68.99	16.37	11.42	5.20	9.12	68.62	2335.00	[51]
Suwałki (Polska)	70,000.00	215.97	142.84	17.04	17.50	63.59	194.78	11,692.80	W tym badaniu

**Table 3:** Pearson’s correlations matrix for the metal concentrations. Correlation is significant at the 0.05 level.

	Cr	Cu	Ni	Zn	Pb	Fe	Mn	OM	pH	Cars per Day
Cr	1.00									
Cu	0.58	1.00								
Ni	0.30	0.44	1.00							
Zn	0.31	0.54	0.20	1.00						
Pb	0.15	0.50	0.57	0.36	1.00					
Fe	0.53	0.71	0.58	0.35	0.45	1.00				
Mn	−0.18	−0.08	0.15	0.09	0.22	−0.18	1.00			
OM	−0.13	−0.05	−0.00	0.18	0.10	−0.06	0.46	1.00		
pH	−0.25	0.09	0.13	−0.08	0.07	−0.16	0.22	−0.13	1.00	
cars per day	0.50	0.64	0.06	0.32	0.22	0.38	−0.34	−0.09	−0.01	1.00

**Table 4:** The rotated component matrix for data of metals in street dusts of Suwalki (n = 25). Principal factors > 0.6 are selected in each column.

	Factor 1	Factor 2
Cr	0.63	−0.43
Cu	0.89	−0.16
Ni	0.67	0.28
Zn	0.63	0.10
Pb	0.70	0.37
Fe	0.81	−0.19
Mn	0.02	0.85
OM	0.05	0.61
pH	−0.01	0.32
cars per day	0.57	−0.49
% of variance	35	19

## Data Availability

Data set available on request to corresponding author.
